# Strophanthus Hispidus

**Published:** 1887-11

**Authors:** L. Deniau, F. A. Harrington


### Translations.

### *STROPHANTHUS HISPIDUS.*

By Dr. L. DENIAU.

Translated from *Bulletin Gen. de Therapeutique*, by F. A. Harrington, A. M., M. D.

Strophanthus hispidus is a member of the botanical family *Apocynaceæ.* It is indigenous to southern Asia, but grows in greatest abundance in tropical Africa. In Africa, certain of the natives employ the plant to poison their arrow-heads. Its active principle is *strophanthine*, a glucoside.

The plant had been the subject of study previously, but it was reserved for Prof. Thos. R. Fraser, of Edinburgh, to point out in detail its chemical, physiological and pharmacological properties, and to make a therapeutic application of the same. His latest account of the drug was published in the *British Medical Journal*, November 14, 1885.

Experiments prove that strophanthus, like digitalis, is a powerful cardiac stimulant, and, if exhibited in sufficient doses, causes death by paralysis of the heart.

After laying bare the heart of a frog poisoned by strophanthus, it will be observed that at first there is increased rapidity of the pulsations, but soon they slacken and finally cease. It is a curious phenomenon that, after the ventricular action stops, the auricles still continue their contraction and relaxation for a brief period.

Not only frogs, but turtles, snails, birds, rabbits, dogs and cats are killed by the active principle of strophanthus. Death ensues sooner after a subcutaneous injection of the drug than by absorption from stomach or rectum.

Experimentation, with a view to compare the rapidity of action of strophanthus and digitalis, establishes the fact that strophanthine produces death much more rapidly than the same dose of the purest crystals of digitaline. In studying the comparative strength of these drugs, it was ascertained that, although a solution of digitaline, one to four thousand, does not stop the heart of a frog, yet a solution of strophanthine, one to ten million, kills the animal.

In warm-blooded animals, death occurs much sooner, and the higher in the scale of life the animal is, the more speedy is the action of strophanthus in destroying life.

In mammals, death is very rapid. In five minutes after the injection of a poisonous dose of strophanthus subcutaneously, you notice signs of *malaise*, dyspnœa, disturbances of respiration, afterwards emesis, muscular paralysis, extreme irregularity of pulse and respiration, and, after a violent convulsion, death.

Susceptibility to the action of the drug may be diminished. As in the case of opium, a therapeutic dose may be taken with impunity.

This dose may be increased gradually until an amount may be administered that, if given at the outset, would have produced toxic effects.

Strophanthus, when introduced into the system, communicates its toxic properties to the blood, as is evidenced when a portion of the blood of a poisoned frog is injected into a healthy animal. Africans realize this fact, for they remove carefully the tissues around the wound in the dead animal.

A small dose has no apparent influence on the motor excitability of nerves, nor on their power of conductivity; and, should the reflexes be abolished, that disappearance is attributable to modifications undergone by the muscles, as they are sooner affected by strophanthus than the nervous system. Unstriped as well as striated muscular fibres are paralyzed, and, when their contractibility is once lost, no stimulus can excite them. The unstriped muscles lose their excitability much more rapidly than striated.

The vascular system is not altered by strophanthus, a characteristic distinguishing it from digitalis.

It has been proven that the drug acts on the heart, not through the medium of the medulla, the sympathetic system, nor the inherent ganglionic centers of the heart. It acts by simple contact with the muscular structure of the heart.

The heart, after death, is in a state of ventricular systole, and its muscle in a condition resembling cadaveric rigidity. It may be noted here that, among all the cardiac poisons, strophanthus leaves the heart after death in systole.

So much for the history and toxicology of strophanthus hispidus. The pharmacology of the plant has a more practical side.

It was in incompetency due to valvular lesions that strophanthus was first proven of inestimable benefit.

Dr. Deniau illustrates, by a series of sphygmographic tracings, the action of strophanthus.

A patient was admitted to the hospital with imperfect systole, orthopnœa, pulmonary œdema, cough, anasarca, passive congestion of the liver and the spleen, cardiac hypertrophy, a soft, indistinct *souffle* of apex, radial pulse, well-nigh imperceptible and incapable of count, and irregular. Under strophanthus, the pulse diminished to forty-eight and respiration to twenty-eight at the end of the fifth day, and there was an amelioration of all the other symptoms.

At the same time, there was a notable increase in the amount of urine, although strophanthus has no action in the calibre of the arteries The diuresis was owing to the stimulation of the heart itself.

Not only in a systole consequent on valvular lesions, but in any case in which a cardiac stimulant is indicated, strophanthus is beneficial.

It has been employed in fatty degeneration of the heart, in acute endocarditis, in atheroma of the arteries, in chronic Bright's disease, in ascites produced by cirrhosis of the liver, and certain pelvic tumors, in the enfeebled heart after acute and chronic fevers, in acceleration of the pulse, and in reflex palpitation of neurasthenia, hysteria and chlorosis.

Strophanthus acts similarly to digitalis in the heart, but is not an accumulative poison. Dr. Emil Pius, of Vienna, says, in disturbances of compensation, strophanthus acts well. The pulse becomes stronger and diminished in frequency, respiration normal, and dyspnœa less marked. In asthma, the paroxysm was shortened and prevented. Diuresis begins, and œdema disappears, not to re-appear save in exceptional cases. In every way, the patient experiences relief.

What is very noteworthy, is that the time in which strophanthus acts is short. Dyspnœa may be relieved in a few minutes, and, in less than an hour, the pulse becomes modified.

The action is persistent. When there is but a slight disturbance of compensation, relief by a single dose continues for three or four months without a relapse.

The drug is especially efficacious against the dyspnœa, orthopnœa, and dropsy of morbus Brighü. Its uremic complications disappear on account of the acid reaction of strophanthus, and the power of increasing the blood pressure without any vaso-motor constriction of the arterioles.

In certain disturbances of circulation, it is of great importance to preserve the patency of the arterial system. In aortic stenosis and insufficiency, and in atheroma of the arteries, it is very essential not to increase the work of the heart by a resistance antagonistic to its propulsive power. The same is true of mitral valvular lesions, and, in a general way, in all diseases accompanied by an increase of the arterial tension.

In almost every instance, it is theoretically advantageous to localize the action of the drug on the heart itself, to the exclusion of the arterial system and the stomach. Strophanthus fully accomplishes this object, and so it is invaluable in the long list of cardiac tonics.

Like digitalis, strophanthus is absolutely contra-indicated in the period of compensation of the heart. It then could only disturb its physiological action. In the feeble heart of pneumonia and phthisis, strophanthus is useful, and Dr. Fraser is inclined to believe that it diminishes the fever.

Thus far there have been only moderately good results in asthma, cardiac neuroses, *agina pectoris*, and in the dyspnœa of hysteria and chlorosis. In a general way, we may state that no good result follows the use of strophanthus in bronchial asthma, nor in acites depending on stasis of the *portal* circulation.

Consequently, strophanthus is contra-indicated in ascites of tumors, hepatic, splenic and pelvic, in respiratory and circulatory troubles of vaso-motor origin, in active hyperæmia, and in cases in which there is a tendency to visceral hemorrhages.

To strophanthus belongs the superiority over digitalis in the treatment of Bright's disease, and, in a general way, in valvular lesions of the heart. As a diuretic, it is not equal to either calomel, digitalis, or acetate of potash.

Thus we have in strophanthus one of the most important acquisitions to therapeutics. It does not exclude digitalis, but one is the complement of the other.

It differs from digitalis in stimulating the heart without any vaso-motor constriction of the arterioles. It diminishes the pulse, lowers temperature somewhat, is a diuretic, is not hæmostatic nor cumulative, and, unlike digitalis, it causes no gastro-intestinal disturbance.

